# The Microtomist's Vade-Mecum

**Published:** 1897-09

**Authors:** 


**The Microtomist's Vade-Mecum.** By ARTHUR BOLLES LEE. Fourth Edition. Pp. xii., 536. London: J. & A. Churchill. 1896.

The fourth edition of this well-known book has been revised throughout, and very many interesting additions have been made. The author has sifted all the new methods suggested during the last three years, and gives us a critical estimate of their value and capabilities. For instance, there is a long account of Weigert's copper hæmatoxylin stain for the central nervous system, and of the many modifications proposed, in which each method is so clearly described that there cannot be the least difficulty in employing it. Again, a little later on, we find the most complete account in the English language of Golgi's silver stains. And we would especially call attention to the explanation of the ripening of logwood stains and of the methods by which such ripening may be obtained in a few seconds of time.

This book has so long occupied the unique position of being the best treatise on the *technique* of microscopic anatomy, that it is superfluous to praise it or to recommend it to workers in that department of science. It is essential both to our practical and theoretical knowledge of the subject.

